# Effects of 8,000 IU aXa long-term prophylaxis with certoparin on the incidence of hyperkalemia in patients with coronary heart disease – a post-hoc analysis of the PARAT trial

**DOI:** 10.1186/1756-0500-7-880

**Published:** 2014-12-06

**Authors:** Nima Melzer, Peter Bramlage, Hans-Christoph Michaelis

**Affiliations:** Novartis Pharma GmbH, Nürnberg, Germany; Institut für Pharmakologie und Präventive Medizin, Menzelstrasse 21, 15831 Mahlow, Germany

**Keywords:** Hyperkalemia, Certoparin, Aldosterone, Renal impairment

## Abstract

**Background:**

Hyperkalemia is an infrequent but potentially serious complication of low molecular weight heparin (LMWH) use. While there are a number of trials comparing LMWH to unfractionated heparin (UFH) there is no comparison of the risk with LMWH versus placebo. Aim of the present post-hoc analysis of the PARAT trial was the description of serum potassium levels with certoparin compared to placebo.

**Results:**

PARAT was a double-blind, placebo-controlled, randomized trial in patients with coronary artery disease receiving either 8,000 I.U. aXa per day or placebo. Serum potassium was monitored at baseline and at scheduled follow-up visits at 2 and 4–6 weeks and 3 and 4–6 months. Statistical evaluation included paired, two sided t-test for each of the treatment groups to compare baseline and follow-up values. A total of 117 patients (59 certoparin, 58 placebo) were included with a mean age of 59 years and 84.6% male gender. There was a statistically significant increase in serum potassium at two weeks after discharge compared to baseline (p < 0.001) in either group which remained elevated throughout the three months treatment phase. Differences between treatment groups were not statistically significant. After treatment discontinuation at the three months’ visit serum potassium returned to normal values (p = n.s. vs. baseline) in both groups. Overall 12 out of 59 patients receiving certoparin (20.3%) and 11 out of 58 patients receiving placebo (19.0%) experienced hyperkalemia based on threshold of >5.0 mmol/l at any time during the observation.

**Conclusions:**

We conclude that there is no incremental risk of hyperkalemia with certoparin up to 8,000 I.U. aXa per day versus placebo in patients with coronary artery disease. The increase in serum potassium values in either group calls for clinical surveillance and the consideration of further risk factors predisposing to hyperkalemia.

## Background

Low-molecular-weight heparins (LMWH) are preferred over unfractionated heparins (UFH) because of the increased bioavailability, less frequent dosing intervals and a reduced need for monitoring. Major adverse events of both heparin types relate to bleeding complications and thrombocytopenia.

Hyperkalemia has been reported to be a rare adverse event of heparin treatment, an increase of serum potassium > 5 mmol/l having been observed in 7% of patients receiving UFH and 15% among those receiving LMWH [1]. It has been described to be a consequence of (reversible) aldosterone suppression, which occurs within a few days after treatment initiation [2–4]. This side effect is more common in the elderly, in patients with renal insufficiency and in those with diabetes mellitus [5]. More recent data report an increase of serum potassium with either heparin type (LMWH or UFH) with no statistically significant differences between groups [6]. Identified risk factors for an increase in potassium during follow-up were potassium levels at baseline, serum creatinine, and creatinine clearance [6].

While there are data on bemiparin [7], nadroparin [2] and enoxaparin [2, 6, 8] insufficient data are available for long-term use and in patients with coronary artery disease. Furthermore there is no placebo controlled trial and hyperkalemia has up to date not been described for certoparin use from clinical trials. Therefore we did a post-hoc analysis of a randomized trial on the use of the low molecular weight heparin versus placebo in patients undergoing coronary angioplasty [9] aiming to describe serum potassium levels with certoparin use and to compare these to placebo.

## Methods

This was a post-hoc analysis of the *Prophylaxis Against Restenosis Angioplasty Trial* (PARAT) which was a double-blind, placebo-controlled, randomized trial with two study groups conducted at four medical centres, the results of which having been previously reported [9]. The protocol was approved by the institutional review boards of each institution. Patients referred for elective balloon angioplasty were asked to participate and to provide written informed consent. Only balloon angioplasty was specified in order to avoid confounding treatment methods. Angiography was repeated at the end of a 6-months observation period.

### Inclusion and exclusion criteria

Patients were required to be at an age between 21 and 80 years, have significant coronary artery disease (CAD, defined as stenosis > 50%), and be able to give informed consent. Excluding conditions were congestive heart failure, other major illnesses (i.e., cancer, liver disease, renal failure, etc.), severe hypertension, child bearing potential, coronary artery bypass surgery (CABG) within 6 weeks, oral anticoagulation therapy, active ulcer or gastrointestinal bleeding, thrombocytopenia, coagulopathy, severe osteoporosis, any cerebral vascular accident (CVA) or transient ischemic attack (TIA), severe diabetic retinopathy, hypersensitivity to heparin or LMWH, or participation in a clinical drug trial within the last 4 weeks.

### Randomization and drug treatment

After coronary angioplasty, patients were monitored at the coronary care unit for 12–36 hours. UFH infusion was initiated at 1,000 I.U. per hour and continued for an additional 12–24 hours to maintain the activated parital thromboplastin time (aPTT) between 60–80 seconds or an activated clotting time (ACT) of 250–300 seconds. Then, patients were randomly assigned to treatment with subcutaneous injections of LMWH (certoparin 8,000 I.U. antiXa) or placebo for 3 months. Thirty minutes after terminating UFH, the first dose of study medication was applied by the study nurse coordinator subcutaneously between 6 and 10 am on the first day after percutaneous coronary intervention (PTCA) who then supervised the self-administered injections until the patient was discharged. Thereafter, over a period of three months, patients had to self administer a subcutaneous injection of certoparin 8000 I.U. aXa or placebo equivalent every morning between 8 and 10 am.

All patients received aspirin (325 mg) daily for the entire study period. Otherwise routine medical treatment of the patients was not altered. Concomitant medications for the treatment of dyslipidemia, diabetes (oral and insulin) had to be documented as drug classes (yes/no), but no specific medication was recorded.

### Investigations

The baseline investigations included chest x-ray, electrocardiogram, bone density scan, urinalysis, stool for occult blood, complete blood count, electrolytes (including serum potassium, blood urea nitrogen [BUN] and creatinine), liver function tests, prothrombin time (PT), aPTT, lipid profile, lipoprotein, lipase, alpha-lipoprotein A and B, and several other investigational measurements relating to thrombosis. Patients were seen by the study coordinators at 2 weeks, 4–6 weeks, 3 months, and 6 months after the angioplasty.

### Statistics and procedures

A total of 170 patients had to be enrolled to meet the study aims as outlined previously [9]. Statistical evaluation included paired, two sided t-test for each of the treatment groups to compare baseline and follow-up values. The potassium plasma levels were measured by routine repetitive medical laboratory analyses using automated analyser systems.

## Results

The study was terminated before the target goal of 170 patients was completed. The reason for the termination was declining enrollment for simple balloon angioplasty in the face of the introduction of coronary atherectomy and stents. Therefore a total of 118 patients with 158 lesions treated with angioplasty were enrolled. For 117 patients data on serum potassium throughout months 0 to 6 were available and 102 patients completed the study and had a follow-up quantitative coronary angiography. Seven patients withdrew and refused to continue. Three patients were withdrawn by their physicians because of new medical conditions which developed. Two patients were discontinued because of hematuria. Four patients were lost to follow-up.

Patients had a mean age of 59 years, 84.6% were male and the majority (n = 103) of caucasian origin. Patients in either group were comparable with respect to age, gender and race (Table 1). The distribution of vessels treated during angioplasty showed that the left anterior descending coronary artery (LAD) was more frequently treated in the LMWH group.

**Table 1:** **Baseline characteristics**

		Certoparin (n = 59)	Placebo (n = 58)
Age (years)	Mean ± SD	60.0 ± 10.0	57.5 ± 11.2
	Min	41	31
	Max	78	79
Gender (%)	Male	83.1	86.2
Race	Caucasean	52	51
	Black	5	7
	Hispanic	2	0
K+ at baseline (mmol/l)	Mean ± SD	4.2 ± 0.4	4.3 ± 0.4
	Min	3.4	3.5
	Max	5.4	5.0
Diabetes mellitus (%)		28.8	19.0
Blood urea (mmol/l)	Mean ± SD	16.5 ± 5.3	16.0 ± 5.6
	Min	7.0	7.0
	Max	31.0	37.0
Serum creatinine (mg/dl)	Mean ± SD	1.1 ± 0.2	1.1 ± 0.3
	Min	0.7	0.7
	Max	1.6	2.0
eGFR (ml/min)	Mean ± SD	95.7 ± 35.5	97.5 ± 32.7
	Min	43.2	41.81
	Max	208.06	186.48

**Figure 1:**
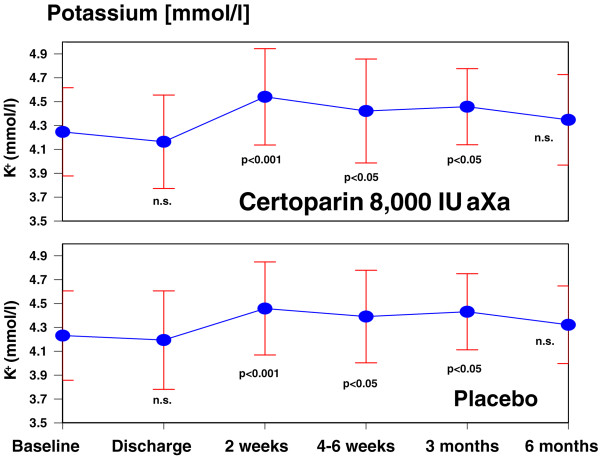
**Serum potassium (mean ± standard deviation) with certoparin or placebo.** Compared to baseline, there is a statistically significant increase of serum potassium in the treatment period in either group. Legend: n.s., non significant; the statistical evaluation was performed using paired, two sided t-test for each of the treatment groups to compare baseline and follow-up values.

Serum potassium values at baseline and for the follow-up visits are shown in Figure 1. There was a statistically significant increase in serum potassium at two weeks after discharge compared to baseline (p < 0.001) in either group which remained elevated throughout the three months treatment phase (p < 0.05 vs. baseline). Differences between treatment groups were not statistically significant (Table 2). Overall 12 out of 59 patients receiving certoparin (20.3%) and 11 out of 58 patients receiving placebo (19.0%) experienced hyperkalemia based on threshold of >5.0 mmol/l. After treatment discontinuation at the three months visit serum potassium returned to normal values (p = n.s. vs. baseline) in both groups.

**Table 2:** **Potassium values**

	Mean ± SD (mmol/l)			> 5 to ≤5.5 mmol/l (%)		> 5.5 mmol/l (%)*	
	Certo	Plac	p-value**	Certo	Plac	Certo	Plac
Baseline	4.2 ± 0.4	4.2 ± 0.4	p = n.s.	3.5	0	0	0
Discharge	4.2 ± 0.4	4.2 ± 0.4	p = n.s.	3.9	4	0	0
2 weeks	4.5 ± 0.4	4.5 ± 0.4	p = n.s.	8.3	6	0	2
4-6 weeks	4.4 ± 0.4	4.4 ± 0.4	p = n.s.	8.9	4.3	0	2.2
3 months	4.4 ± 0.3	4.5 ± 0.3	p = n.s.	4.8	4.4	0	0
6 months	4.3 ± 0.3	4.3 ± 0.4	p = n.s.	4.9	2.4	0	0

*Legend:* *there were no patients with a serum potassium > 6.0 mmol/l; **the statistical evaluation was performed using paired, two sided t-test for each of the treatment groups to compare baseline and follow-up values.

## Discussion

First case reports of patients with heparin induced hyperkalemia date back to 1980 where Phelps and colleagues published the case of a 77 year old man with a creatinine clearance of 23 to 27 ml/min who developed hyperkalemia while receiving heparin for peripheral arterial insufficiency [10]. Discontinuation of heparin led to resolution of hyperkalemia as the plasma aldosterone concentration multiplied six-fold. Since then a total of 6 clinical studies [1, 2, 7, 8, 11, 12] have been published on this issue (as to a PubMed search dated September 2014 with the keywords heparin & hyperkalemia and the restriction “clinical trial”, the latest being the work of Torres et al. in May 2010 reflecting the continued clinical implication of this rare but relevant complication [7].

Heparin-induced increases in serum potassium are mediated by a reversible effect of heparin on aldosterone leading to hypoaldosteronism [2, 3]. It is effected by both a reduction of the number and affinity of angiotensin II receptors in the zona glomerulosa [4]. Prolonged heparin application has been shown to result in a reduced width of the adrenal zona glomerulosa, an effect that appears to be more pronounced with UFH than LMWH [12, 13]. Available studies on LMWH induced hyperkalemia have usually compared incidence rates with those observed with UFH [6, 12, 14] or were purely observational [7, 15]. These studies suggested that hyperkalemia rates with LMWH were as high [6] or even lower [12] than those with UFH. No study has however reported rates for LMWH as compared to placebo. Our own post-hoc analysis of a trial involving patients undergoing coronary angioplasty revealed no significant differences between the LMWH certoparin (at 8,000 I.U. aXa per day) and placebo without any significant differences in serum potassium and hyperkalemia over a three months period. The different propensity of LMWH and UFH to result in hyperkalemia is potentially important in our analysis because there was a UFH run-in period of 12–24 hours after coronary angioplasty, after which patients were randomized to receive certoparin or placebo. This run-in period was, however, rather short compared to the time course of hyperkalemia development in different analyses [1], suggesting (but not excluding) no interference of UFH with the later potassium values.

There was an increase of serum potassium within the two weeks after hospital discharge however that might have been precipitated by adjuvants present in the solution for injection or the prescription of drugs inducing hyperkalemia in either group such as ACE inhibitors or spironolactone or the discontinuation of diuretics. The role of concomitant treatments is actually the result of a previous report demonstrating that baseline potassium and concomitant angiotensin converting enzyme (ACE) inhibitor use [7] predicted the development of hyperkalemia. We were not able to verify this since detailed data on concomitant drug treatment (neither specific drugs not doses) were not obtained.

Our results are in principal agreement with a prior observational study by Abdel-Raheem who reported no major increase in serum potassium with 2×40 mg enoxaparin per day [15]. It is in partial contrast, however, to a warning in the summary of product characteristics (SPCs) of enoxaparin and certoparin that potassium should be monitored in patients with increased serum potassium and those with a high risk. The SPC of tinzaparin even tells that serum potassium has to be monitored in short intervals.

The results are, of course, of particular relevance for the population under investigation in the PARAT trial. These were characterized by a referral for elective balloon angioplasty. They had significant coronary artery disease and 41% prior myocardial infarction. Further frequent concomitant conditions were hypertension (50.0%), diabetes (13.6%), prior PTCA (20.3%) and prior CABG (7.6%). In addition these patients frequently present with risk factors for the development of hyperkalemia such as impaired kidney function and often receive a prescription of drugs increasing serum potassium levels in its own (spironolactone and ACE inhibitors). Therefore the population under investigation represent a high-risk population for the development of hyperkalemia and lesser rates can be expected for patients receiving LMWH overall.

### Limitations

There are a number of limitations for this analysis that need consideration: 1) The sample size was rather small with 107 being available for the potassium analyses in both groups combined. At a rate of hyperkalemia of 20.3% for certoparin and 19.0% for placebo several thousand patients would be necessary to determine a statistically significant difference. Even in this case the clinical value of such a difference may be limited. 2) Concomitant medication may interfere with the risk of hyperkalemia up and beyond heparin or placebo. In the CRF only the presence of drugs for the treatment of dyslipidemia and diabetes (oral and insulin) were recorded, but no specific drugs nor those in particular with an interaction potential on serum potassium.

## Conclusions

We conclude that there is no incremental risk of hyperkalemia with certoparin up to 8,000 I.U. aXa per day versus placebo in patients with coronary artery disease. However the increase in serum potassium values after hospital discharge in either group call for clinical surveillance and the consideration of further risk factors predisposing to hyperkalemia such as renal impairment, diabetes mellitus and concomitant pharmacotherapy such as ACE inhibitors and aldosterone antagonists.
